# RNA Processing Factors Swd2.2 and Sen1 Antagonize RNA Pol III-Dependent Transcription and the Localization of Condensin at Pol III Genes

**DOI:** 10.1371/journal.pgen.1004794

**Published:** 2014-11-13

**Authors:** Pénélope Legros, Amélie Malapert, Sho Niinuma, Pascal Bernard, Vincent Vanoosthuyse

**Affiliations:** CNRS, Université Lyon 01, UMR5239, LBMC; Ecole Normale Supérieure de Lyon, Lyon, France.; The University of North Carolina at Chapel Hill, United States of America

## Abstract

Condensin-mediated chromosome condensation is essential for genome stability upon cell division. Genetic studies have indicated that the association of condensin with chromatin is intimately linked to gene transcription, but what transcription-associated feature(s) direct(s) the accumulation of condensin remains unclear. Here we show in fission yeast that condensin becomes strikingly enriched at RNA Pol III-transcribed genes when Swd2.2 and Sen1, two factors involved in the transcription process, are simultaneously deleted. Sen1 is an ATP-dependent helicase whose orthologue in *Saccharomyces cerevisiae* contributes both to terminate transcription of some RNA Pol II transcripts and to antagonize the formation of DNA:RNA hybrids in the genome. Using two independent mapping techniques, we show that DNA:RNA hybrids form in abundance at Pol III-transcribed genes in fission yeast but we demonstrate that they are unlikely to faciliate the recruitment of condensin. Instead, we show that Sen1 forms a stable and abundant complex with RNA Pol III and that Swd2.2 and Sen1 antagonize both the interaction of RNA Pol III with chromatin and RNA Pol III-dependent transcription. When Swd2.2 and Sen1 are lacking, the increased concentration of RNA Pol III and condensin at Pol III-transcribed genes is accompanied by the accumulation of topoisomerase I and II and by local nucleosome depletion, suggesting that Pol III-transcribed genes suffer topological stress. We provide evidence that this topological stress contributes to recruit and/or stabilize condensin at Pol III-transcribed genes in the absence of Swd2.2 and Sen1. Our data challenge the idea that a processive RNA polymerase hinders the binding of condensin and suggest that transcription-associated topological stress could in some circumstances facilitate the association of condensin.

## Author Summary

Failure to condense chromosomes prior to anaphase onset can lead to genome instability. The evolutionary-conserved condensin complex drives chromosome condensation, probably by changing the topology of chromatin around its binding sites. Condensin localizes to regions of high transcription, suggesting that some transcription-associated feature(s) direct its association with chromatin. Here we considered that transcription-dependent DNA:RNA hybrids or topological stress could be involved in recruiting condensin. Our data show that condensin is indeed enriched at regions accumulating DNA:RNA hybrids but that they are not involved in its recruitment. Rather, we identify a mutant combination where increased transcription by RNA Pol III is associated locally with stronger topological stress. Strikingly the localization of condensin is dramatically enhanced at the same loci and we show that topological stress contributes to this enhanced association. Our data strengthen the idea that transcription creates the environment necessary to recruit condensin in mitosis.

## Introduction

Mitotic chromosome condensation is essential for genome integrity. When defective, chromosomes often remain entangled and fail to segregate properly in anaphase. A key driver of chromosome condensation is the highly conserved condensin complex. Condensin is made of five sub-units (SMC2^Cut14^, SMC4^Cut3^, CAP-D2^Cnd1^, CAP-G^Cnd3^ and CAP-H^Cnd2^, name of the human protein followed by its name in fission yeast) and it is one of the main components of mitotic chromosomes [1]. *In vitro*, purified condensin can introduce positive supercoils into a relaxed plasmid in the presence of topoisomerase I [2], [3]. These observations support the idea that condensin shapes mitotic chromosomes by changing the topology of chromatin around its binding sites. However, the mechanisms underlying the association of condensin with chromatin remain poorly understood (reviewed in [4]).

Several studies have illustrated the paradoxical relationships linking gene transcription and the localization of condensin. From pro- to eukaryotes, condensin is preferentially enriched at highly transcribed genes [5], [6], [7], [8], suggesting that some highly conserved transcription-associated feature(s) that predate(s) the appearance of nucleosomes help to recruit condensin. However, experiments in yeast indicated that RNA polymerases must be silenced before condensin can bind, at least at repetitive sequences such as the rDNA or the sub-telomeres [9], [10]. These somewhat contradictory observations could potentially be reconciled if one hypothesizes that a by-product of the transcription process facilitates the recruitment of condensin. In this study, we have considered that such a by-product could be R-Loops or transcription-associated topological stress.

R-Loops result from the formation of stable DNA:RNA hybrids in the genome. As a consequence of the hybridization of the RNA to the template, the non-transcribed strand of the DNA remains single-stranded (reviewed in [11]). Interestingly, the hinge domain of the Smc2/Smc4 heterodimer in condensin shows high affinity *in vitro* for single-stranded DNA [12], [13]. Moreover, a recent study proposed that chromatin is less accessible to restriction enzymes in mutants where R-Loops accumulate, consistent with the idea that R-Loop formation favours chromatin compaction [14]. Interestingly, fission yeast condensin can disassemble DNA:RNA hybrids *in vitro* [15] and its chicken counterpart localizes to CpG islands [6], which constitute major R-Loop forming regions in the genome [16]. Taken together, these observations support the idea that R-Loops and condensin could interact functionally *in vivo* [14].

According to the twin supercoiled domain model, high rates of transcription induce positive supercoiling of the chromatin in front of the elongating polymerase, whilst negative supercoiling accumulate upstream of the polymerase [17]. As such, highly expressed genes represent regions of the genomes that accumulate topological stress. As confirmed *in vivo* recently, this stress is monitored by topoisomerase I and topoisomerase II [18], [19], [20]. Interestingly, *in vitro* assays have indicated that condensin binds preferentially to positively supercoiled plasmids in the presence of ATP [21]. Whether or not this transcription-associated topological stress contributes to the binding of condensin *in vivo* has not been addressed.

In order to clarify the functional relationships between transcription and chromosome condensation, we recently carried out a genetic screen in fission yeast to identify deletions of transcription-associated factors that would rescue a condensin deficiency [22]. For this, we isolated loss-of-function mutations that could rescue the thermo-sensitivity of the condensin mutant *cut3-477* [23]. Two of the mutations we isolated were the deletions of *swd2.2* (*swd2.2Δ*) and *sen1* (*sen1Δ*) [22]. Swd2.2 is a non-essential component of the Cleavage and Polyadenylation Factor (CPF), the complex responsible for 3′end maturation of RNA Pol II transcripts in yeast (reviewed in [24]), where it acts to maintain the proper levels of CPF-associated phosphatases [22]. Fission yeast Sen1 is the homologue of human Senataxin and has been shown to unwind DNA:RNA hybrids *in vitro* [25]. Budding yeast Sen1 is involved in transcription termination [26] but its role in fission yeast has not been characterized. Here we show that both factors act directly at Pol III-transcribed genes to limit the association of condensin and the accumulation of topological stress. Furthermore, topological stress at Pol III-transcribed genes facilitates the association of condensin when Swd2.2 and Sen1 are missing.

## Results

### Swd2.2 and Sen1 negatively regulate the accumulation of condensin at Pol III-transcribed genes

**Figure 1:**
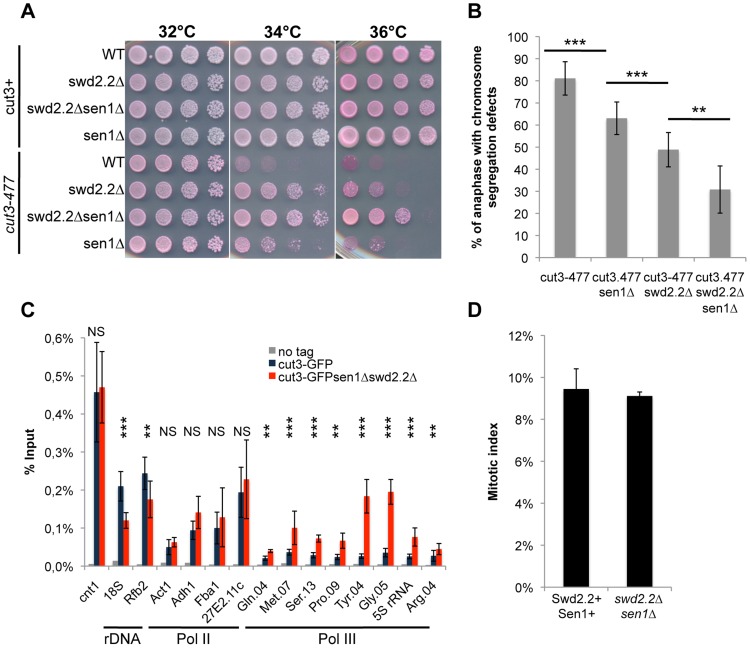
The double deletion of Swd2.2 and Sen1 facilitates the localization of condensin at Pol III-transcribed genes. **A**. Serial dilutions of the indicated strains were plated on rich media at the indicated temperatures. **B**. Chromosome segregation in anaphase was monitored in the indicated strains after growing cells for one generation at 34°C. For each genotype, a minimum of 6 independent experiments was performed in which a minimum of 100 anaphase cells were scored (***<0.001; **<0.01 Wilcoxon - Mann Whitney). Anaphases were scored as defective when chromatin was detected lagging between the two main DNA masses **C**. ChIP-qPCR analysis of the amount of GFP-tagged Cut3 cross-linked to chromatin in cell populations of the indicated genotypes grown at 30°C (mean ± standard deviation from 6 biological replicates (NS: not significant, *P<0.05; **P<0.01; ***P<0.001 Wilcoxon - Mann Whitney). The primers used in this study are shown on Table S1. **D**. Mitotic indexes of the cell populations used in **C**. Cells were fixed with cold methanol and processed for immuno-fluorescence using an anti-tubulin antibody. Cells with a spindle were counted as mitotic.

On their own, the deletions of *swd2.2* (*swd2.2Δ*) and *sen1* (*sen1Δ*) partly restored growth of *cut3-477* cells at the restrictive temperature (Figure 1A) and reduced the proportion of anaphase cells displaying chromosome segregation defects (Figure 1B). Combining both deletions (*sen1Δswd2.2Δ*) resulted in a stronger suppressor effect (Figure 1AB). The double mutant *sen1Δswd2.2Δ* also suppressed the other condensin mutant *cut14-208* (Figure S1). Strikingly, Chromatin Immunoprecipitation (ChIP) analysis in cycling cell populations showed that the localization of condensin was altered at specific loci when Swd2.2 and Sen1 were both missing: its recruitment increased significantly at genes transcribed by RNA Pol III (Gln.04, Met.07, Ser.13, Pro.09, Tyr.04, Gly.05, 5S rRNA, Arg.04 on Figure 1C), whereas it was significantly reduced at the rDNA arrays (18S&Rfb2). The binding of condensin remained unaffected at kinetochores (cnt1) or at highly transcribed Pol II genes (Act1, Adh1, Fba1 and SPAC27E2.11c). The sequences of all the primers used in this study are available on Table S1. The mitotic indexes of both cell populations (swd2.2+sen1+ and *swd2.2Δsen1Δ*) were comparable (Figure 1D), ruling out that the changes in the association of condensin are due to indirect, cell-cycle defects. These data established that Sen1 and Swd2.2 act to limit the localization of condensin at Pol III-transcribed genes. The reasons why the association of condensin at the rDNA arrays is reduced in the absence of Swd2.2 and Sen1 will be explained elsewhere.

### Swd2.2 and Sen1 localize at Pol III-transcribed genes and regulate the transcription cycle of RNA Pol III

**Figure 2:**
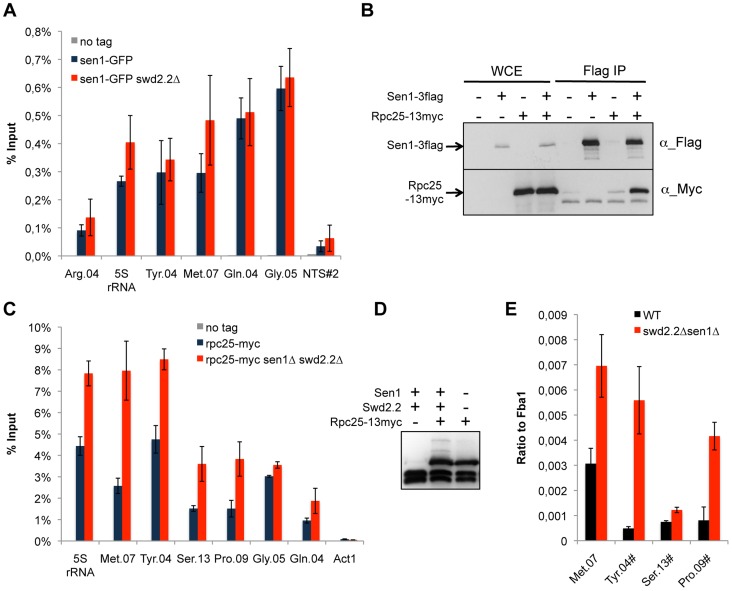
Transcription is enhanced at Pol III-transcribed genes when Swd2.2 and Sen1 are missing. **A**. Sen1 is enriched at Pol III-transcribed genes. ChIP qPCR of the indicated strains grown in cycling conditions at the indicated loci (mean ± standard deviation from 3 biological replicates). NTS#2 is a site within the Replication Fork Barrier of the rDNA and is shown as a comparison. **B**. Flag-tagged Sen1 co-immunoprecipitates with Myc-tagged Rpc25. Whole cell extracts (WCE) and the immuno-precipitated material (Flag IP) of the indicated strains were analyzed by western blot. **C**. Rpc25 becomes more abundant at Pol III-transcribed genes when Swd2.2 and Sen1 are missing. ChIP qPCR of the indicated strains grown in cycling conditions at the indicated loci (mean ± standard deviation from 3 biological replicates). **D**. Western blot analysis of the stability of Rpc25-13myc. Tubulin is used as a loading control. **E**. Pol III transcripts are more abundant when Swd2.2 and Sen1 are missing. Total RNAs extracted from swd2.2+sen1+ or *swd2.2Δsen1Δ* cells grown in rich medium at 30°C were analyzed by RT-qPCR (3 biological replicates, 2 RT per replicate).

We found previously that Swd2.2 associates with Pol III-transcribed genes and that lack of Swd2.2 restored the localization of condensin at Pol III-transcribed genes in the condensin-deficient mutant *cut3-477* [22]. Here, we show that Sen1 is also significantly enriched at Pol III-transcribed genes and that its binding is independent of Swd2.2 (Figure 2A). Furthermore, affinity purification of Sen1 followed by mass-spectrometry analysis of its associated proteins identified most sub-units of the RNA Pol III complex as its most stable binding partners (Table S2). We confirmed this interaction by showing that the RNA Pol III sub-unit Rpc25 co-precipitates with Sen1 (Figure 2B). Note however that Sen1 did not co-precipitate with Sfc6, a sub-unit of TFIIIC (Figure S2), a complex required for the association of RNA Pol III with chromatin [27]. ChIP analysis showed that the association of Rpc25 with chromatin was significantly increased in the absence of Sen1 (Figure S3) or in *swd2.2Δsen1Δ* cells (Figure 2C&D). In *swd2.2Δsen1Δ* cells, the stabilization of RNA Pol III on chromatin was associated with an increase in the steady-state level of tRNAs, as detected by RT-qPCR analysis (Figure 2E). Taken together, these experiments concur to show that Swd2.2 and Sen1 play a direct role at Pol III-transcribed genes, where they limit the association of RNA Pol III and the accumulation of transcripts. These results show that the accumulation of condensin at Pol III-transcribed genes in *swd2.2Δsen1Δ* cells is concomitant with an enhanced transcriptional activity.

### R-Loops accumulate strongly at Pol III-transcribed genes

It was recently argued that budding yeast Sen1 limits the accumulation of DNA:RNA hybrids, including at Pol III-transcribed genes [28]. Fission yeast Sen1 similarly was shown to display a DNA:RNA helicase activity *in vitro* [25]. These observations and the additional arguments detailed in the introduction prompted us to test the possibility that R-Loops could represent a transcription by-product facilitating the association of condensin with chromatin. We speculated that lack of Sen1 and Swd2.2 could result in the accumulation of R-Loops at Pol III-transcribed genes where they might contribute to increase the association of condensin.

**Figure 3:**
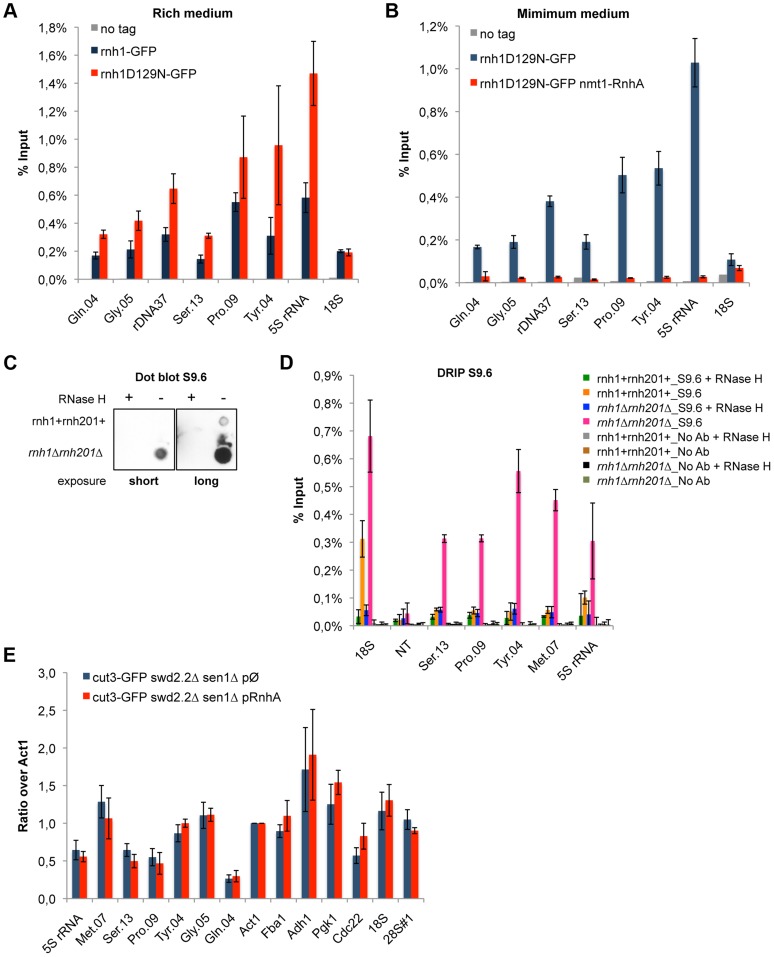
R-Loops form in abundance at Pol III-transcribed genes but they do not significantly impact the association of condensin. **A**. ChIP qPCR of the indicated strains grown in cycling conditions at the indicated loci (mean ± standard deviation from 3 biological replicates). **B**. As in **A**. Cells were grown in minimal medium for a minimum of 18 hours to promote the over-expression of RnhA driven by the nmt promoter. **C**. Genomic DNA was extracted from rnh1+rnh201+ and *rnh1Δrnh201Δ* cells in preparation for the DRIP procedure. Equal amount of genomic DNA were spotted on a nylon membrane and incubated with 2 µg/mL of purified S9.6 antibody. The amount of S9.6 bound to the DNA was revealed using chemiluminescence. **D**. DRIP-qPCR of the indicated strains grown in cycling conditions at the indicated loci (mean ± standard deviation from 3 biological replicates). **E**. Cells of the indicated genotypes were grown in minimum medium lacking thiamine for a minimum of 18 hours to drive the over-expression of RnhA. ChIP-qPCR was then performed (mean ± standard deviation from 3 biological replicates).

To establish whether or not R-Loops form at Pol III-transcribed genes in fission yeast, we first monitored by ChIP the chromatin association of RNase H1, one of the endogenous enzymes known to disassemble R-Loops. More specifically, we introduced at the endogenous locus a point mutation (D129N) in the fission yeast RNase H1 (Rnh1), because the same mutation was shown to weaken the catalytic activity of human RNase H1 [29]. Consistent with this, the D129N mutation did stabilize the interaction of Rnh1 with Pol III-transcribed genes (Figure 3A). Furthermore, the interaction of Rnh1D129N with Pol III-transcribed genes was lost upon over-expression *in vivo* of RnhA, the RNase H1 enzyme from *E.coli* (Figure 3B). Upon over-expression, RnhA itself did not stably associate with Pol III-transcribed genes (Figure S4), showing that the loss of Rnh1D129N from Pol III-transcribed genes upon over-expression of RnhA cannot be explained by its mere replacement by bacterial RnhA. Finally, Figure S5 shows that the association of Rnh1D129N with the rDNA repeats increased significantly in the absence of topoisomerase I (*top1Δ*), consistent with the observations reported previously that lack of Top1 triggers the accumulation of R-Loops at rDNA in budding yeast [30]. This confirmed that Rnh1D129N was able to detect significant changes in R-Loop accumulation. Taken together, these data show that ChIP with Rnh1D129N is a reliable way to identify R-Loop forming regions in fission yeast.

We sought to confirm the formation of R-Loops at genes transcribed by RNA Pol III using another approach. A method that is commonly used to map R-Loop forming regions in yeast is to perform ChIP using the S9.6 antibody because of its high affinity for DNA:RNA hybrids [31]. ChIP requires formaldehyde cross-linking followed by sonication of the chromatin. We found that the ability of S9.6 to detect R-Loops generated after transcription *in vitro* was greatly diminished both by formaldehyde cross-linking and by sonication (Figure S6). We do not know at this stage whether this is because R-Loops are partly destroyed by these treatments or because these treatments reduce the affinity of the antibody for R-Loops. To circumvent these issues, we extracted genomic DNA from unfixed cells, digested soluble RNA using RNase A and sheared the DNA using a cocktail of restriction enzymes (see Methods). Dot blot analysis using the S9.6 antibody confirmed that our procedure largely preserved R-Loops (Figure 3C). We then performed DNA:RNA immuno-precipitation (DRIP) using the S9.6 antibody in stringent conditions, in the presence of 500 mM NaCl. As expected, the DRIP signal at 18S, the canonical R-Loop forming region within the rDNA repeats [30], increased significantly in the absence of RNase H1 and RNase H2 (*rnh1Δrnh201Δ* cells) and disappeared almost entirely upon treatment of the genomic DNA with commercial RNase H (Figure 3D). On the contrary, the DRIP signal detected at a non-transcribed region NT (chr I, 3009300-3009500, [32]) remained low both in *rnh1Δrnh201Δ* cells and upon treatment with RNase H. Those controls demonstrated that the signals we detected using DRIP were specific. In agreement with the results obtained using ChIP of Rnh1D129N as a reporter for the presence of R-Loops, we detected strong DRIP signals at Pol III-transcribed genes in the absence of RNase H1 and RNase H2 (Figure 3D). In conclusion, the two methods we have set up to map R-Loop forming regions establish that R-Loops are a prominent feature of Pol III-transcribed genes in fission yeast.

Using ChIP of Rnh1D129N, we established that R-Loops accumulate to similar levels at Pol III-transcribed genes in cycling cells (>90% of interphase cells) and in cells synchronized in early mitotis (Figure S7A). Consistent with this, ChIP established that the association of RNA Pol III with chromatin is largely maintained in mitosis (Figure S7B). Taken together these experiments support the idea that transcription at Pol III-transcribed genes is maintained in mitosis, at a time when condensin is loaded on chromosomes in fission yeast.

Finally, lack of Swd2.2 and Sen1 resulted in a small but significant increase in the formation of R-Loops at some but not all Pol III-transcribed genes (Figure S8). Note however that this increase could be due to the fact that Pol III transcription is stimulated in the absence of Swd2.2 and Sen1 (Figure 2C&D). As such, these observations therefore do not prove that Swd2.2 and Sen1 antagonize R-Loop formation at Pol III-transcribed genes directly.

### Stable R-Loop formation is not necessary to recruit condensin

To establish whether R-Loops at Pol III-transcribed genes could contribute to the accumulation of condensin, we prevented the formation of stable R-Loops by over-expressing RnhA. ChIP analysis showed that over-expression of RnhA did not reduce the amount of condensin recruited at Pol III-transcribed genes in *swd2.2Δsen1Δ* cells (Figure 3E) or in wild-type mitotic cells (Figure S9). These data concur to demonstrate that stable, long-lived R-Loops play little or no part in recruiting condensin. Note that over-expression of RnhA did not interfere either with the association of RNA Pol III (Figure S10A) or Sen1 (Figure S10B).

### Topological constraints accumulate in cells lacking Swd2.2 and Sen1

**Figure 4:**
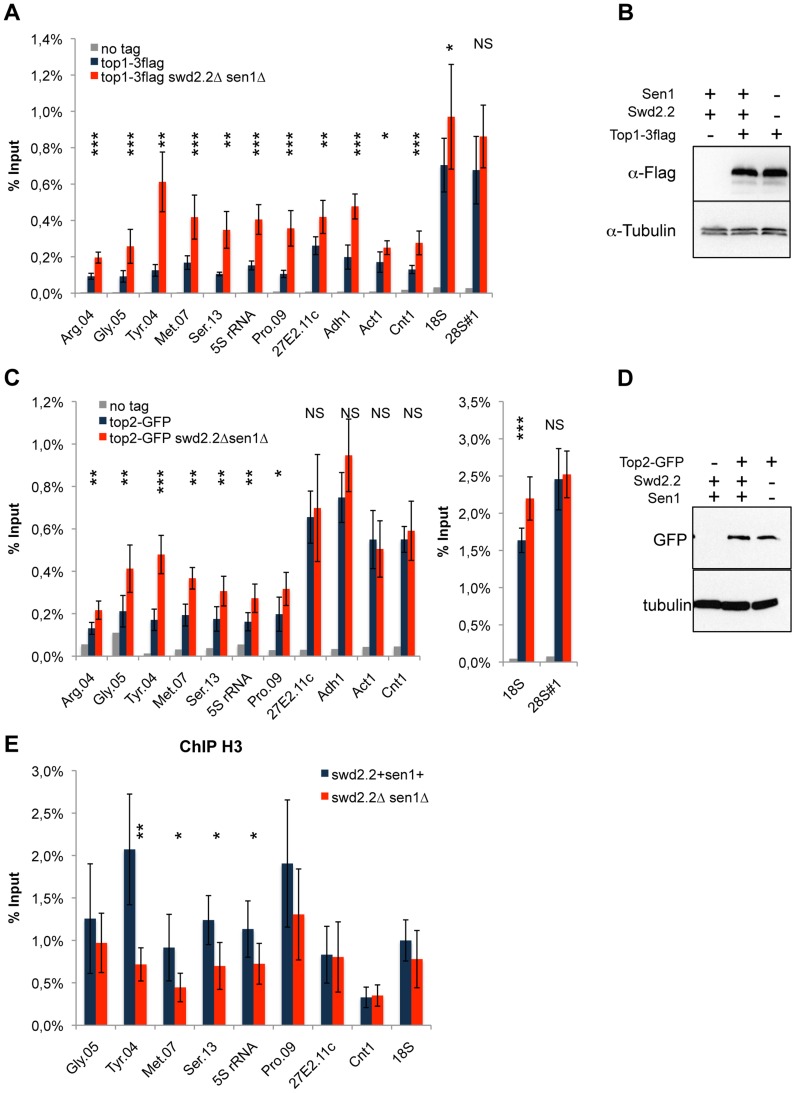
Lack of Swd2.2 and Sen1 results in local topological stress at Pol III-transcribed genes. **A**. ChIP qPCR of the indicated strains grown in cycling conditions at the indicated loci (mean ± standard deviation from 6 biological replicates. NS: not significant *P<0.05; **P<0.01; ***P<0.001 Wilcoxon - Mann Whitney). **B**. Western blot analysis of the stability of Top1-3flag. Tubulin is used as a loading control. **C**. ChIP qPCR of the indicated strains grown in cycling conditions at the indicated loci (mean ± standard deviation from 6 biological replicates. NS: not significant *P<0.05; **P<0.01; ***P<0.001 Wilcoxon - Mann Whitney). **D**. Western blot analysis of the stability of Top2-GFP. Tubulin is used as a loading control. **E**. ChIP qPCR of histone H3 in the indicated strains grown in cycling conditions at the indicated loci (mean ± standard deviation from 6 biological replicates. NS: not significant *P<0.05; **P<0.01; Wilcoxon - Mann Whitney).

Because Xenopus condensin shows greater affinity *in vitro* for positively supercoiled DNA [21], we speculated that the cue facilitating the accumulation of condensin at Pol III-transcribed genes in the absence of Swd2.2 and Sen1 could be local topological constraints. Consistent with an increase in topological stress in *swd2.2Δsen1Δ* cells, ChIP analysis detected strong accumulation of topoisomerase I (Top1) at most loci (Figure 4A), although the protein levels of Top1 remained unaffected (Figure 4B). We also detected enhanced accumulation of topoisomerase II (Top2), mostly at Pol III-transcribed genes (Figure 4C), when the protein levels of Top2 remained unaffected (Figure 4D). Transcription-associated topological stress was recently shown to destabilize nucleosomes [19]. At some but not all Pol III-transcribed genes that we tested, we detected a significant reduction in the recruitment of histone H3 (Figure 4E) in *swd2.2Δsen1Δ* cells, which is consistent with the local depletion of nucleosomes. The concomitant accumulation of Top1 and Top2 and the depletion of nucleosomes suggest that topological stress is greater at Pol III-transcribed genes in *swd2.2Δsen1Δ* cells. We speculate that the increased transcription of Pol III-transcribed genes in *swd2.2Δsen1Δ* cells could contribute at least in part to this enhanced topological stress.

As R-Loops unwind the DNA, it was possible that the abundance of R-Loops formed at Pol III-transcribed genes (Figure 3) could contribute to this topological stress. To test this possibility, we monitored by ChIP the localization of Top2 upon over-expression of RnhA. Surprisingly, the localization of Top2 was not altered at Pol III-transcribed genes upon over-expression of RnhA, whilst it was reduced at the Pol I-transcribed 18S (Figure S11). This suggested that the impact of R-Loop formation on the surrounding chromatin depends on where in the genome R-Loops form.

### Topological stress contributes to the loading of condensin at Pol III-transcribed genes in the absence of Swd2.2 and Sen1

**Figure 5:**
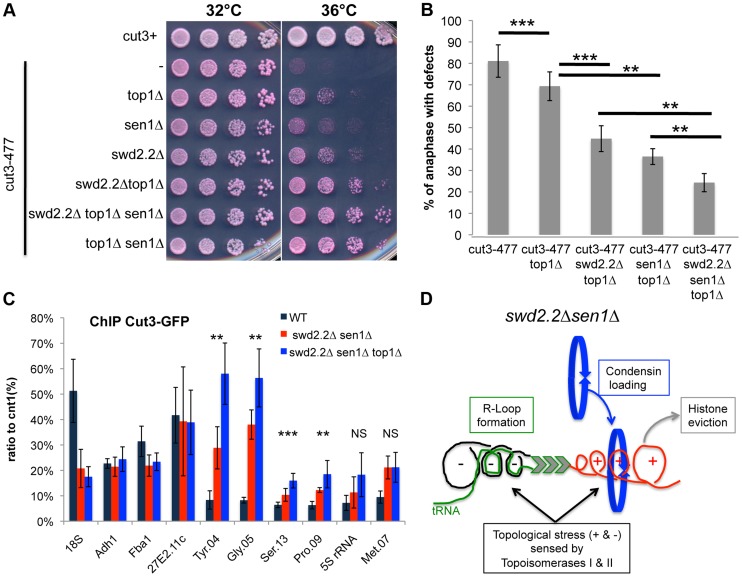
Lack of Top1 further increases the association of condensin with Pol III-transcribed genes when Swd2.2 and Sen1 are missing. **A**. Serial dilutions of the indicated strains were plated on rich media at the indicated temperatures. **B**. Chromosome segregation in anaphase was monitored in the indicated strains after growing cells for one generation at 34°C. For each genotype, a minimum of 6 independent experiments was performed in which a minimum of 100 anaphase cells were scored (***<0.001; **<0.01 Wilcoxon - Mann Whitney). Anaphases were scored as defective when chromatin was detected lagging between the two main DNA masses **C**. ChIP qPCR of the indicated strains grown in cycling conditions at the indicated loci (mean ± standard deviation from 6 biological replicates. NS: not significant *P<0.05; **P<0.01; ***P<0.001 Wilcoxon - Mann Whitney). **D**. Model. Lack of Swd2.2 and Sen1 increases gene transcription at Pol III-transcribed genes. According to the twin supercoiled domain model, this results in more positive supercoils downstream of the polymerase and compensatory negative supercoils upstream of the polymerase. Negative supercoils favor the formation of R-Loops (reviewed in [41]). Positive supercoils result in nucleosome eviction. This topological stress also facilitates the recruitment of condensin, either directly or indirectly.

Based on these results, we envisaged two possible models to explain the increased localization of condensin at Pol III-transcribed genes in the absence of Swd2.2 and Sen1: either the accumulation of Top1 and/or Top2 helps to recruit and/or stabilize condensin, or topological stress facilitates the association of condensin at Pol III-transcribed genes. We previously identified the deletion of Top1 (*top1Δ*) as a suppressor of *cut3-477* [22], suggesting that the accumulation of Top1 that results from lack of Swd2.2 and Sen1 is unlikely to facilitate the association of condensin with chromatin. Figures 5A&B show that the triple deletion *swd2.2Δsen1Δtop1Δ* was a better suppressor of *cut3-477* than the double deletion *swd2.2Δsen1Δ*. This genetic evidence suggested that failure to monitor topological stress in *top1Δ* cells might facilitate the association/function of condensin. In support of this, ChIP analysis showed that there was a small but significant increase in the association of condensin at most Pol III-transcribed genes in cells deleted for Swd2.2, Sen1 and Top1 (*swd2.2Δsen1Δtop1Δ* cells) (Figure 5C). Taken together, these data support the following model: the absence of Swd2.2 and Sen1 increases the transcriptional activity at Pol III-transcribed genes and this might contribute to enhance local topological constraints. These constraints, either directly or indirectly, contribute to recruit or maintain condensin at Pol III-transcribed genes (Figure 5D).

### Topological stress is not sufficient to recruit condensin

To establish whether topological stress was sufficient to stimulate the association of condensin with chromatin, we monitored the association of condensin in the temperature-sensitive Top2 mutant *top2-191* ([33]) at the semi-restrictive temperature of 28°C. This analysis showed that the association of condensin was not significantly disrupted in these conditions (Figure S12A). Similarly, lack of Top1 on its own did not significantly impact the association of condensin (Figure S12B). Taken together, these observations suggest that topological stress on its own is not sufficient to stimulate the association of condensin with chromatin.

## Discussion

### Topological stress facilitates the recruitment of condensin at Pol III-transcribed genes in the absence of Swd2.2 and Sen1

In order to explain that condensin localizes to highly expressed genes from pro- to eukaryotes, whatever the RNA polymerase involved, we first hypothesized that a transcription by-product could facilitate the association of condensin with chromatin (see Introduction). We speculated that this mechanism could represent the ancestral way of recruiting condensin to chromatin. Complementary *cis*-acting factors would then have evolved to stabilize the interaction of condensin with specific loci, as shown previously (reviewed in [4]).

In this study we specifically considered two transcription by-products as potential condensin-attracting features: R-Loop formation and transcription-associated topological stress. Both features have been described both in pro- and eukaryotes and they generate structures (single-stranded DNA and positive supercoiling) for which condensin has been shown to display high affinity *in vitro*. Our data are not consistent with the idea that stable R-Loops could be involved in recruiting condensin. Similarly, topological stress on its own was not sufficient to disrupt the localization pattern of condensin. However, our data show that topological stress facilitated the association of condensin at Pol III-transcribed genes when Swd2.2 and Sen1 were missing. These observations are consistent with the recent demonstration that supercoiling at highly expressed genes contributes to the establishment of topological domains and small-range chromosome compaction in *Caulaboacter crescentus* [34].

How could topological stress create a better binding site for condensin at Pol III-transcribed genes in the absence of Swd2.2 and Sen1? First, condensin might simply have a higher affinity for supercoiled chromatin, as suggested by the observation that condensin associates preferentially *in vitro* with positively supercoiled plasmids [21]. Alternatively, or in addition, topological stress might work by facilitating nucleosome eviction [19]. Consistent with the latter, budding yeast condensin associates preferentially with nucleosome-free regions, especially at Pol III-transcribed genes [12]. To explain that lack of Top1 only facilitates the association of condensin at Pol III-transcribed genes when Swd2.2 and Sen1 are missing, we speculate that the level of topological stress has to go over a certain threshold in order to attract/stabilize condensin. This threshold would be reached in the chromatin around Pol III-transcribed genes when Swd2.2 and Sen1 are missing but not when Top1 only is missing.

### Two reliable tools to map R-Loop forming regions in fission yeast

The biology of R-Loops is a rapidly expanding field of investigation, and many observations now demonstrate that R-Loops control genome stability and gene expression in multiple ways (reviewed in [35]). It is therefore essential to establish reliable methods to map R-Loop forming regions in genetically tractable organisms such as yeast to address the many functions of R-Loops *in vivo*. We presented evidence that the commonly used S9.6 ChIP method to map R-Loop forming regions in yeast is challenged by the fact that R-Loops, or at least their recognition by the S9.6 antibody, are partly sensitive to formaldehyde cross-linking and sonication. To circumvent this problem, we have developed two reliable alternatives to map R-Loop forming regions in fission yeast. Both of our methods concur to demonstrate that RNA-Pol III transcribed genes are major R-Loop forming regions in fission yeast. R-Loops have also been detected at Pol III-transcribed genes in budding yeast ([28]), suggesting that R-Loop formation is a conserved feature of Pol III transcription, at least in yeast.

We would like to argue that the two methods we have set up are complementary: not only do they map R-Loop forming regions but their use in parallel can also give information regarding the stability of R-Loops formed at different loci. Our data show that RNase H1 is most abundant at Pol III-transcribed genes throughout the cell-cycle, suggesting that R-Loops are constantly formed and detected by RNase H1 there. Our data also show that over-expression of RnhA *in vivo* counter-acts R-Loop formation more efficiently at Pol III-transcribed genes than within the rDNA for example (18S, Figure 3B). On the contrary, DRIP only yields significant signals at Pol III-transcribed genes when RNase H1 and RNase H2 are missing (*rnh1Δrnh201Δ* cells), whilst the DRIP signals at the rDNA (18S) are significant in wild-type cells, when RNase H1 and RNase H2 are fully active. At Pol III-transcribed genes, DRIP signals increase 10-20 fold in *rnh1Δrnh201Δ* cells, whilst they only increase ∼3-fold at the rDNA (18S). Our interpretation of these data is that R-Loops formed at 18S are stable and a relatively poor substrate for RNase H1, whilst R-Loops formed at Pol III-transcribed genes are unstable and a good substrate for RNase H1. A corollary to these observations is that DRIP is probably better suited to detect long-lived, stable R-Loops. This might explain why DRIP did not detect significant R-Loop formation at Pol III-transcribed genes in human cells ([16], [36]). We conclude that using both R-Loop mapping methods in parallel could provide indications of the relative stability of R-Loops at different loci.

The reasons why R-Loops formed at Pol III-transcribed genes are labile are still unclear but we speculate that R-Loops formed at Pol III-transcribed genes might be smaller than those formed at the 18S because the Pol III transcription units are much smaller. Further studies will be required to understand the consequences of R-Loop formation at Pol III-transcribed genes and how the half-life of an R-Loop might influence its function.

### R-Loop-mediated chromosome compaction versus condensin-mediated chromosome condensation

R-Loop formation has been shown to be associated with increased phosphorylation of histone H3 on Serine 10 and reduced chromatin accessibility [14]. In turn, the phosphorylation of histone H3 on Serine 10 facilitates the interaction between adjacent nucleosomes, thereby promoting chromatin compaction [37]. We showed previously that to constitutively increase the levels of histone H3 phosphorylated on Serine 10 by deleting PP1 phosphatase (*dis2Δ*) was not sufficient to significantly improve chromosome segregation when condensin was deficient [22], suggesting that H3-S10-mediated chromatin compaction cannot compensate for the deficiency of condensin. Here we presented evidence that stable R-Loops do not significantly contribute to the recruitment of condensin. Taken together, these observations concur to establish that R-Loop-mediated chromatin compaction is distinct from condensin-mediated chromosome condensation. Our data also suggest that the action of condensin is more fundamental to building a mitotic chromosome than R-Loop-mediated chromatin compaction.

### RNA processing factors and genome stability

Our data have highlighted unexpected ways by which proteins involved in the metabolism of RNA can affect chromosome segregation and genome integrity. Published data demonstrated conclusively that mutations in such factors in general and in Sen1 in particular resulted in chromosome instability (CIN) in yeast, in a mechanism involving R-Loop formation antagonizing replication fork progression ([38], [39] and reviewed in [35]). Here on the contrary, our data show that deletions of two such factors, Swd2.2 and Sen1, facilitate the segregation and stability of chromosomes when condensin is deficient, in a mechanism that does not require stable R-Loop formation.

In addition, our data show that Swd2.2 and Sen1 keep topological stress under control at Pol III-transcribed genes. We speculate that the enhanced transcription at Pol III-transcription associated with lack of Swd2.2 and Sen1 could contribute to such stress. However, we cannot exclude the possibility that RNA Pol III-dependent transcription is also defective in other ways that could explain the accumulation of topological stress when Swd2.2 and Sen1 are missing. The answer to this question will require further studies.

### Sen1 antagonizes RNA Pol III-dependent transcription in fission yeast

Beautiful *in vitro* approaches demonstrated unequivocally that budding yeast Sen1 contributes to transcription termination of some RNA Pol II transcripts ([26]). It is not yet known whether fission yeast Sen1 has the same function. As fission yeast Sen1 is not essential for viability whilst its budding yeast counterpart is, it is possible that the function of Sen1 has diverged in fission yeast. This idea is supported by our data showing that RNA Pol III is likely to be the most stable binding partner of Sen1 in fission yeast and that Sen1 antagonizes Pol III-dependent transcription. On the contrary, a recent study aimed at identifying the binding partners of RNA Pol III in budding yeast did not identify Sen1, suggesting that the interaction between Sen1 and RNA Pol III is not as stable and/or abundant in budding yeast [40]. Further work is required to understand the function of fission yeast Sen1 at Pol III-transcribed genes.

### Conclusion

Previous studies had concluded that the inhibition of RNA Pol I or RNA Pol II in mitosis was a pre-requisite for the binding of condensin at repetitive sequences [9],[10], suggesting that a processive RNA polymerase is a hindrance to the binding of condensin on chromatin. Here we challenge this idea by showing that an enhanced recruitment of condensin at Pol III-transcribed genes is associated with an increase in the expression of the same genes. These data show that, at least at Pol III-transcribed genes, an active polymerase is not an obstacle for the binding of condensin.

## Materials and Methods

### Fission yeast strains

A complete list of all of the strains used in this study is given in Table S3. Standard genetic crosses were employed to construct all strains. Rnh1-GFP, Sen1-GFP, and Top1-3flag were generated using a standard PCR procedure. To obtain Rnh1D129N, Rnh1 was PCR amplified and cloned into pCRII (Life technologies). Site-directed mutagenesis was then used to mutate the residue D129 into N (GAC to AAC) using Quickchange protocols (Stratagene). Overlapping PCR was used to add a C-terminus GFP tag and a cassette of resistance to kanamycin (KanR) to the mutagenized Rnh1 in order to integrate the mutagenized *Rnh1* at the endogenous *Rnh1* locus. After yeast transformation, proper integrants were selected by PCR and western blot and were sequenced to verify the presence of the mutation. The plasmid over-expressing RnhA tagged with 1xFLAG at its N-terminus was obtained from Eun Shik Choi and Robin Allshire (WTCCB, Edinburgh, UK). In order to stably integrate the plasmid in the genome, it was linearized by digestion with MluI and then transformed in to yeast according to standard procedures.

### Chromatin immunoprecipitation

1,5.10^8^ cells were treated with 1% formaldehyde (Sigma) at 17°C for 30′. After extensive washes with cold PBS, cells were frozen in liquid Nitrogen. Frozen cells were then broken open using a RETSCH MM400 Mill and then resuspended in cold lysis buffer (Hepes-KOH 50 mM pH 7,5, NaCl 140 mM, EDTA 1 mM, Triton 1%, Na-deoxycholate 0,1%, PMSF 1 mM). The lysats were then sonicated at 4°C using a Diagenode sonicator. Immuno-precipitation was done overnight at 4°C using Protein A-coupled Dynabeads previously incubated with the anti-GFP A11122 antibody (Invitrogen) or using Protein G-coupled Dynabeads previously incubated with the anti-myc 9E10 antibody (Sigma) according to the manufacturer's instructions. Beads were washed successively with (5′ incubation on rotating wheel): Wash I buffer (20 mM Tris pH 8, 150 mM NaCl, 2 mM EDTA, 1% Triton-X100, 0,1% SDS), Wash II buffer (20 mM Tris pH 8, 500 mM NaCl, 2 mM EDTA, 1% Triton-X100, 0,1% SDS) and Wash III buffer (20 mM Tris pH 8, 1 mM EDTA, 0,5% Na-deoxycholate, 1% Igepal, 250 mM LiCl). After two additional washes in TE pH 8, the beads were resuspended in 10% Chelex resin (Biorad) and incubated at 98°C for 10′. After addition of 2 µL of 10 mg/mL of proteinase K, the mixture was incubated at 43°C for 1 hour, then for another 10 mn at 98°C. After centrifugation, the supernatant was collected and analyzed by qPCR.

### DRIP

8.10^8^ cells were frozen in liquid nitrogen, broken open using a RETSCH MM400 Mill and then resuspended in cold lysis buffer (Hepes-KOH 50 mM pH 7,5, NaCl 140 mM, EDTA 1 mM, Triton 1%, Na-deoxycholate 0,1%). After phenol/chloroform purification and ethanol precipitation, the DNA was resuspended in TE pH 8 and split into two samples. Both samples were digested with BsrGI, EcoRI, HindIII, SspI and XbaI according to the manufacturer's instructions and RNase H was added to one of the two samples. After digestion, each sample was divided into two and incubated overnight at 4°C in IP buffer (100 mM MES pH 6,6, NaCl 500 mM, 0,05% Triton, 2 mg/mL BSA) in the presence of either Protein A-coupled Dynabeads or Protein A-coupled Dynabeads previously incubated with the S9.6 antibody according to the manufacturer's instructions. The beads were then washed three times in IP buffer. After two additional washes in TE pH 8, the beads were resuspended in 10% Chelex resin (Biorad) and incubated at 98°C for 5′. After addition of 2 µL of 10 mg/mL of proteinase K, the mixture was incubated at 43°C for 30′, then for another 5′ at 98°C. After centrifugation, the supernatant was collected and analyzed by qPCR.

### Immunoprecipitation

Immunoprecipitation was carried out as described previously [22], except that cells were broken open using a RETSCH MM400 Mill. To purify Sen1-associated proteins (Table S2), a protein extract was prepared from 10^9^ cells expressing GFP-tagged Sen1 from the endogenous locus. After immuno-precipitation with 15 µL of magnetic beads, the beads were washed three times with 1 mL of lysis buffer and twice with 1 mL of PBS containing 0,02% Tween. The beads samples were then subjected to in-solution reduction, carbamidomethylation and tryptic digestion. After acidification with 10%Trifluoroacetic Acid the samples were centrifuged 3 times to eliminate the beads.

### Mass-spectrometry analysis

Peptide sequences were determined by mass spectrometry performed using a LTQ Velos instrument (Dual Pressure Linear Ion Trap) equipped with a nanospray source (Thermo Fisher Scientific) and coupled to a U3000 nanoLC system (Thermo Fisher Scientific). A MS survey scan was acquired over the m/z range 400–1600 in Enhanced resolution mode. The MS/MS scans were acquired in Normal resolution mode over the m/z range 65–2000 for the 20 most intense MS ions with a charge of 2 or more and with a collision energy set to 35eV. The spectra were recorded using dynamic exclusion of previously analyzed ions for 0.5 min with 50 millimass units (mmu) of mass tolerance. The peptide separation was obtained on a C18 PepMap micro-precolumn (5 µm; 100 Å; 300 µm×5 mm; Dionex) and a C18 PepMap nanocolumn (3 µm; 100 Å; 75 µm×200 mm; Dionex) using a linear 90 min gradient from 0 to 40%, where solvent A was 0.1% HCOOH in H2O/CH3CN (95/5) and solvent B was 0.1% HCOOH in H2O/CH3CN (20/80) at 300 nL/min flow rate.

Protein identification was performed using the MASCOT Algorithm from the Proteome Discoverer software v1.1 (Thermo Fisher Scientific) against the UniProtKB database reduced to *Schizosaccharomyces pombe* species [UniProt release 2013_12].

### RNA extraction and RT-qPCR

These were performed as previously described [22].

## Supporting Information

Figure S1 Lack of Swd2.2 and Sen1 suppresses the growth defect of the *cut14-208* mutant of condensin. Serial dilutions of the indicated strains were plated on rich media at the indicated temperatures
(TIF)

Figure S2 Sen1 associates with RNA Pol III but not with TFIIIC. Flag-tagged Sen1 co-immunoprecipitates with myc-tagged RNA Pol III component Rpc25 but not with myc-tagged TFIIIC component Sfc6. Whole cell extracts (WCE) and the immuno-precipitated material (Flag IP) of the indicated strains were analyzed by western blot.
(TIF)

Figure S3 The RNA Pol III subunit Rpc25 becomes more abundant at Pol III-transcribed genes when Sen1 is missing. **A**. ChIP-qPCR of the indicated strains grown in cycling conditions, at the indicated loci (mean ± standard deviation from 3 biological replicates). **B**. Western blot analysis of the stability of Rpc25-13myc.
(TIF)

Figure S4 Over-expressed RnhA does not replace the endogenous Rnh1 at Pol III-transcribed genes. ChIP qPCR analysis of the indicated strains grown in minimum medium to drive the over-expression of RnhA by the *nmt1* promoter (mean ± standard deviation from 6 biological replicates). After cross-linking and sonication, the whole cell extract was divided in two. In one half, the endogenous GFP-tagged Rnh1D129N was immuno-precipitated with a GFP antibody. In the other half, the over-expressed Flag-tagged RnhA was immuno-precipitated with a Flag antibody. For comparison, values were normalized to the enrichment obtained at 18S.
(TIF)

Figure S5 R-Loops accumulate at 18S in the absence of Top1. ChIP qPCR of the indicated strains grown in cycling conditions at the indicated loci (mean ± standard deviation from 3 biological replicates).
(TIF)

Figure S6 The amount of R-Loops detected by the S9.6 antibody is reduced after sonication and formaldehyde cross-linking. R-Loops derived from the mouse *AIRN* gene were generated after transcription *in vitro* with the T3 polymerase as described previously [16]. The products of *in vitro* transcription were then digested with RNase A, sonicated for 10′ and/or cross-linked with 1% formaldehyde for 10′. The DNA was purified and either (**A**) run on an agarose gel without Ethidium bromide or (**B**) analyzed by dot-blot using the S9.6 antibody.
(TIF)

Figure S7 R-Loop form at Pol III-transcribed genes in early mitotic cells. nda3+ (cycling) and *nda3KM311* (mitotic) cells were grown at the restrictive temperature of 17°C for 6 hours to synchronize cells prior to anaphase onset. ChIP-qPCR was performed to monitor the association of (**A**) Rnh1D129N-GFP or (**B**) Rpc25-13myc at the indicated loci (mean ± standard deviation from 3 biological replicates).
(TIF)

Figure S8 Lack of Swd2.2 and Sen1 results in a small but significant accumulation of R-Loops at Pol III-transcribed genes. ChIP qPCR of the indicated strains grown in cycling conditions at the indicated loci (mean ± standard deviation from 6 biological replicates. *P<0.05; **P<0.01; ***P<0.001 Wilcoxon - Mann Whitney).
(TIF)

Figure S9 RnhA over-expression does not displace condensin from chromatin in mitotic cells. **A**. Cells carrying the cold-sensitive *nda3-KM311* mutation were grown in minimum medium lacking thiamine for a minimum of 18 hours to drive the over-expression of RnhA by the *nmt1* promoter and then shifted at 17°C for 6 hours to synchronize them prior to anaphase onset. ChIP-qPCR was then performed to analyze the association of GFP-tagged Cut3 with chromatin (mean ± standard deviation from 3 biological replicates). **B**. Mitotic indexes of the cell populations in A as established by scoring the number of Cut3-GFP-positive nuclei.
(TIF)

Figure S10 RnhA over-expression does not impact the association of RNA Pol III or Sen1 at Pol III-transcribed genes. **A**. ChIP qPCR of the indicated strains grown in cycling conditions in minimum medium at the indicated loci (mean ± standard deviation from 3 biological replicates). **B**. ChIP qPCR of the indicated strains grown in cycling conditions in minimum medium at the indicated loci (mean ± standard deviation from 3 biological replicates).
(TIF)

Figure S11 RnhA over-expression impacts the association of Top2 with chromatin differently at the rDNA and at Pol III-transcribed genes. ChIP qPCR of the indicated strains grown in cycling conditions in minimum medium at the indicated loci (mean ± standard deviation from 3 biological replicates).
(TIF)

Figure S12 The association of condensin with chromatin is not significantly altered in the topoisomerase mutants *top2-191* and *top1Δ*. **A** ChIP qPCR of the indicated strains grown in cycling conditions at the semi-restrictive temperature of 28°C at the indicated loci (mean ± standard deviation from 3 biological replicates). **B**. ChIP qPCR of the indicated strains grown in cycling conditions at the semi-restrictive temperature of 30°C at the indicated loci (mean ± standard deviation from 6 biological replicates).
(TIF)

Table S1 Primers used in this study.
(XLSX)

Table S2 Identification by mass-spectrometry analysis of the proteins associated with Sen1 in fission yeast.
(XLSX)

Table S3 Yeast strains used in this study.
(XLSX)
