# Supplementary figures and images for: RNA Processing Factors Swd2.2 and Sen1 Antagonize RNA Pol III-Dependent Transcription and the Localization of Condensin at Pol III Genes
Source: PLoS Genet. 2014 Nov 13;10(11):e1004794. doi: 10.1371/journal.pgen.1004794 (PMC4230746; doi:10.1371/journal.pgen.1004794)

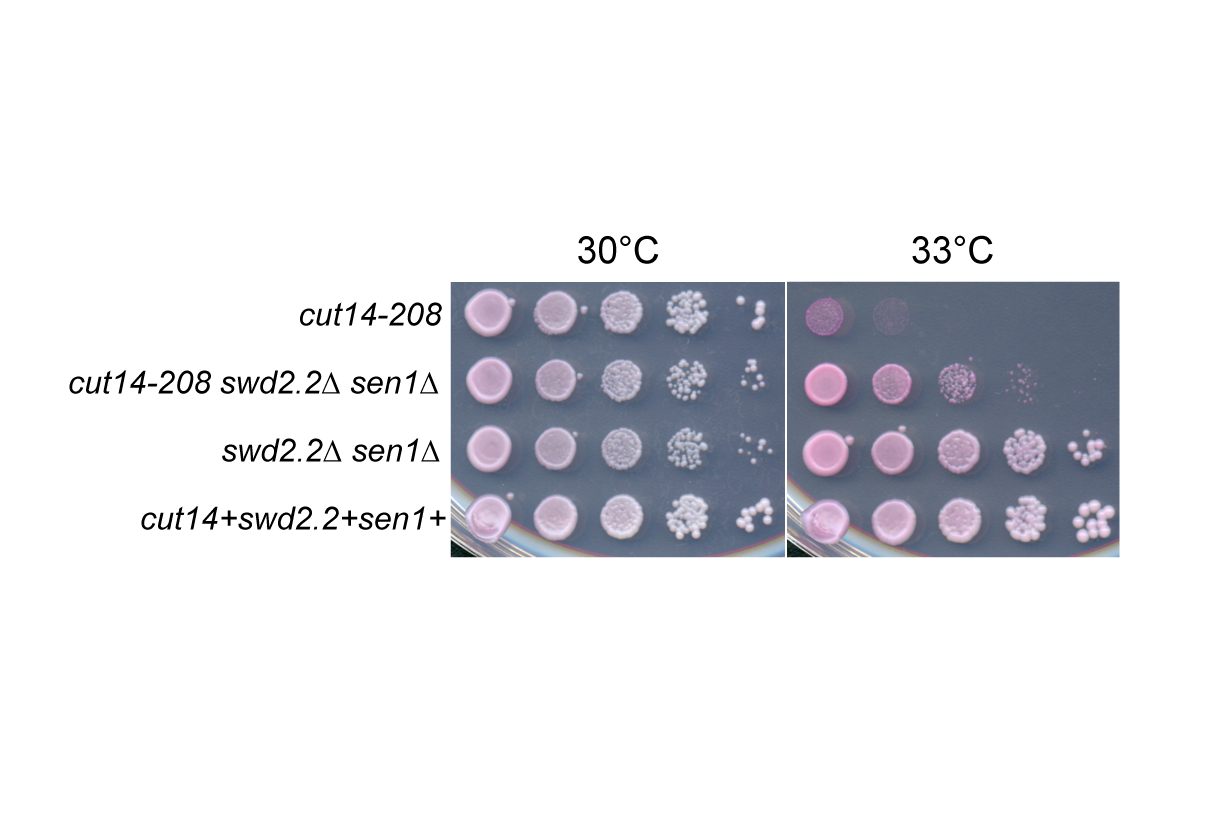

Supplement: Figure S1 — Lack of Swd2.2 and Sen1 suppresses the growth defect of the cut14-208 mutant of condensin. Serial dilutions of the indicated strains were plated on rich media at the indicated temperatures (TIF) [file pgen.1004794.s001.tif]

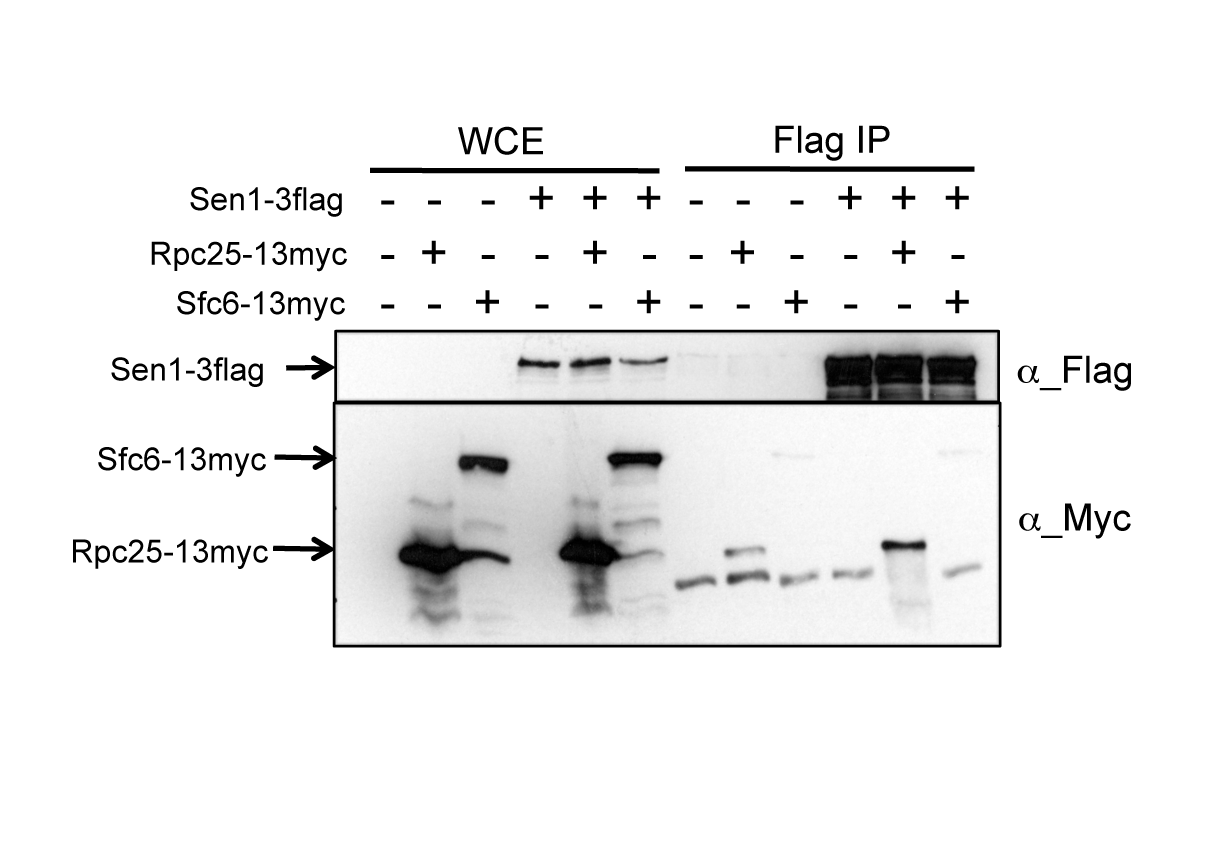

Supplement: Figure S2 — Sen1 associates with RNA Pol III but not with TFIIIC. Flag-tagged Sen1 co-immunoprecipitates with myc-tagged RNA Pol III component Rpc25 but not with myc-tagged TFIIIC component Sfc6. Whole cell extracts (WCE) and the immuno-precipitated material (Flag IP) of the indicated strains were analyzed by western blot. (TIF) [file pgen.1004794.s002.tif]

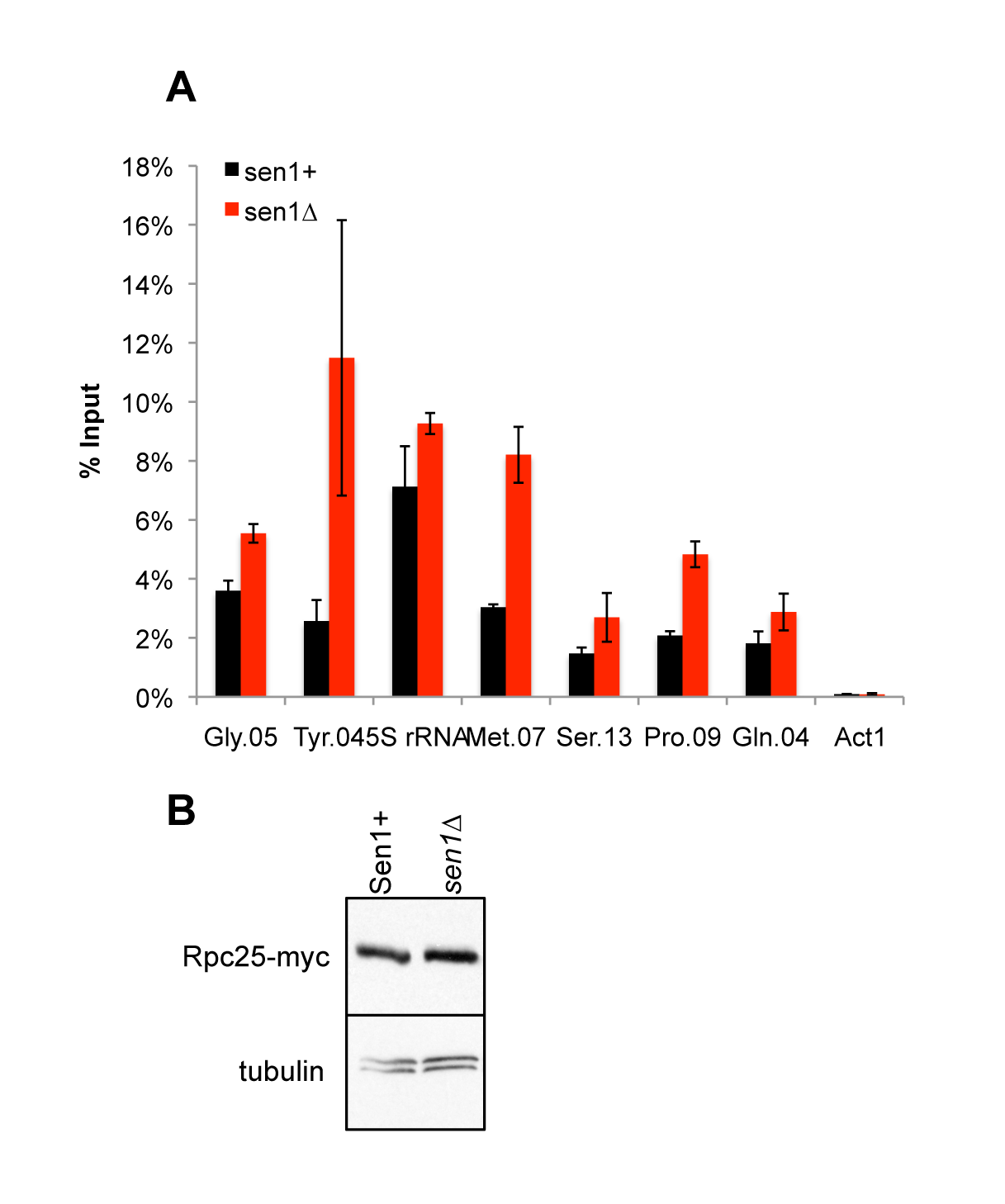

Supplement: Figure S3 — The RNA Pol III subunit Rpc25 becomes more abundant at Pol III-transcribed genes when Sen1 is missing. A. ChIP-qPCR of the indicated strains grown in cycling conditions, at the indicated loci (mean ± standard deviation from 3 biological replicates). B. Western blot analysis of the stability of Rpc25-13myc. (TIF) [file pgen.1004794.s003.tif]

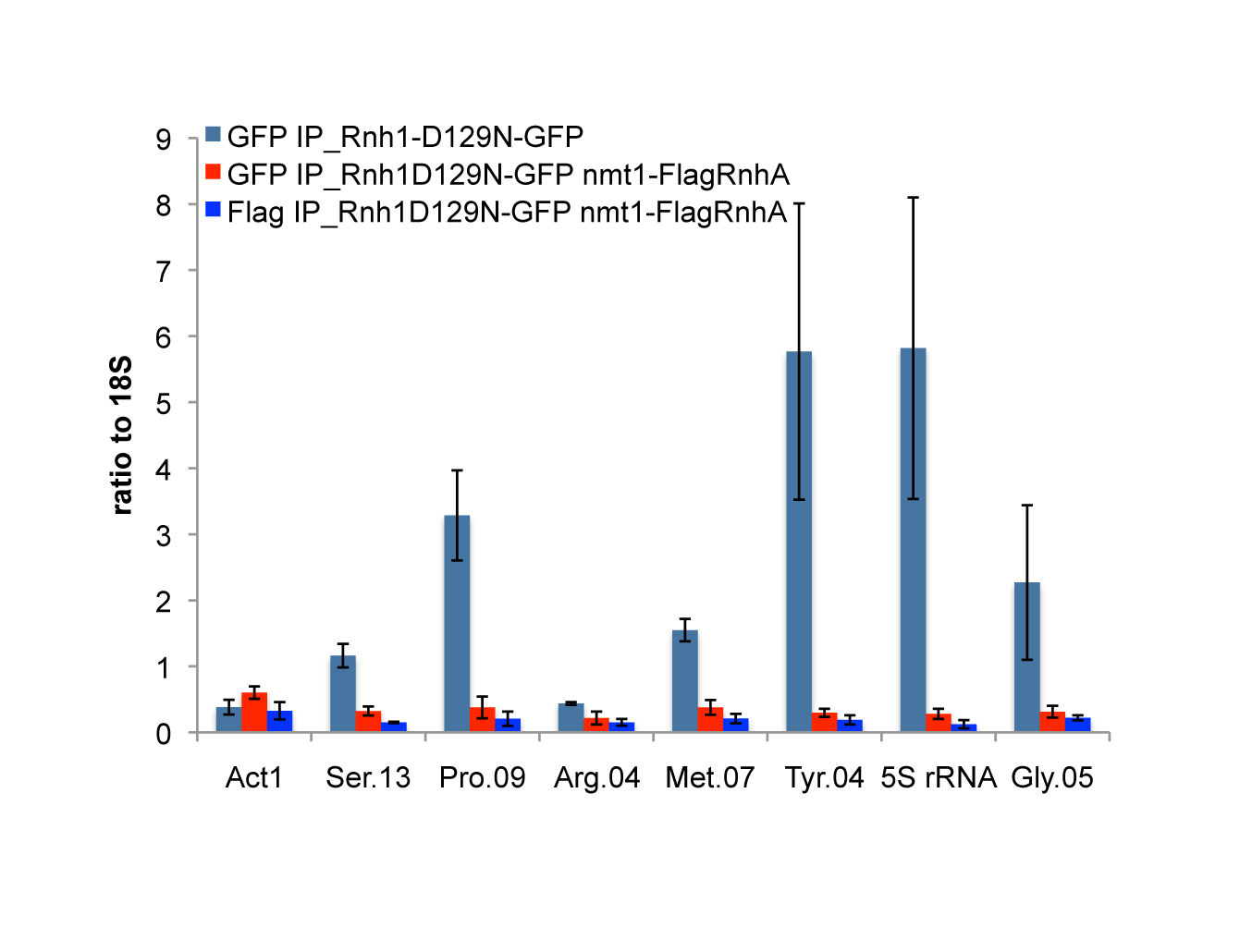

Supplement: Figure S4 — Over-expressed RnhA does not replace the endogenous Rnh1 at Pol III-transcribed genes. ChIP qPCR analysis of the indicated strains grown in minimum medium to drive the over-expression of RnhA by the nmt1 promoter (mean ± standard deviation from 6 biological replicates). After cross-linking and sonication, the whole cell extract was divided in two. In one half, the endogenous GFP-tagged Rnh1D129N was immuno-precipitated with a GFP antibody. In the other half, the over-expressed Flag-tagged RnhA was immuno-precipitated with a Flag antibody. For comparison, values were normalized to the enrichment obtained at 18S. (TIF) [file pgen.1004794.s004.tif]

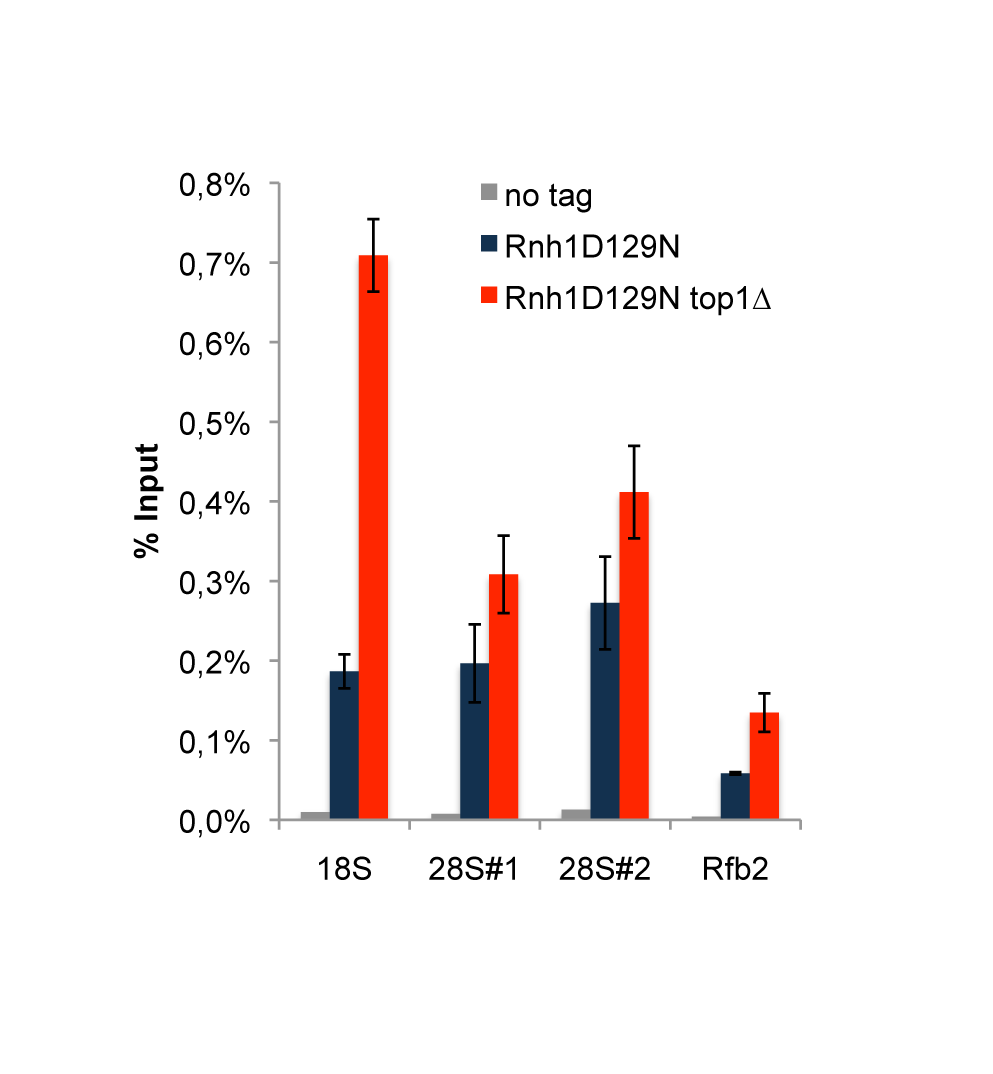

Supplement: Figure S5 — R-Loops accumulate at 18S in the absence of Top1. ChIP qPCR of the indicated strains grown in cycling conditions at the indicated loci (mean ± standard deviation from 3 biological replicates). (TIF) [file pgen.1004794.s005.tif]

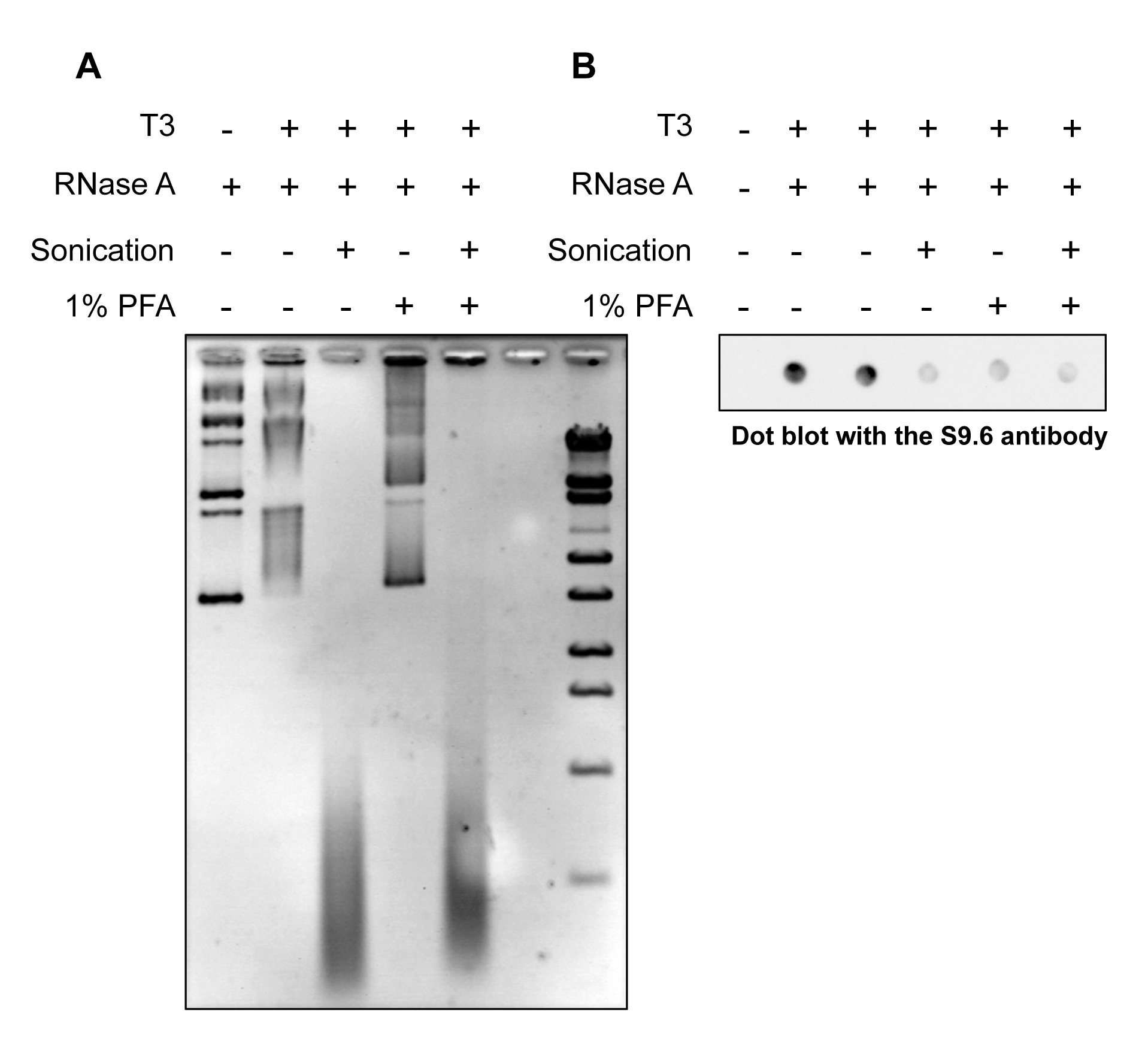

Supplement: Figure S6 — The amount of R-Loops detected by the S9.6 antibody is reduced after sonication and formaldehyde cross-linking. R-Loops derived from the mouse AIRN gene were generated after transcription in vitro with the T3 polymerase as described previously [16]. The products of in vitro transcription were then digested with RNase A, sonicated for 10′ and/or cross-linked with 1% formaldehyde for 10′. The DNA was purified and either (A) run on an agarose gel without Ethidium bromide or (B) analyzed by dot-blot using the S9.6 antibody. (TIF) [file pgen.1004794.s006.tif]

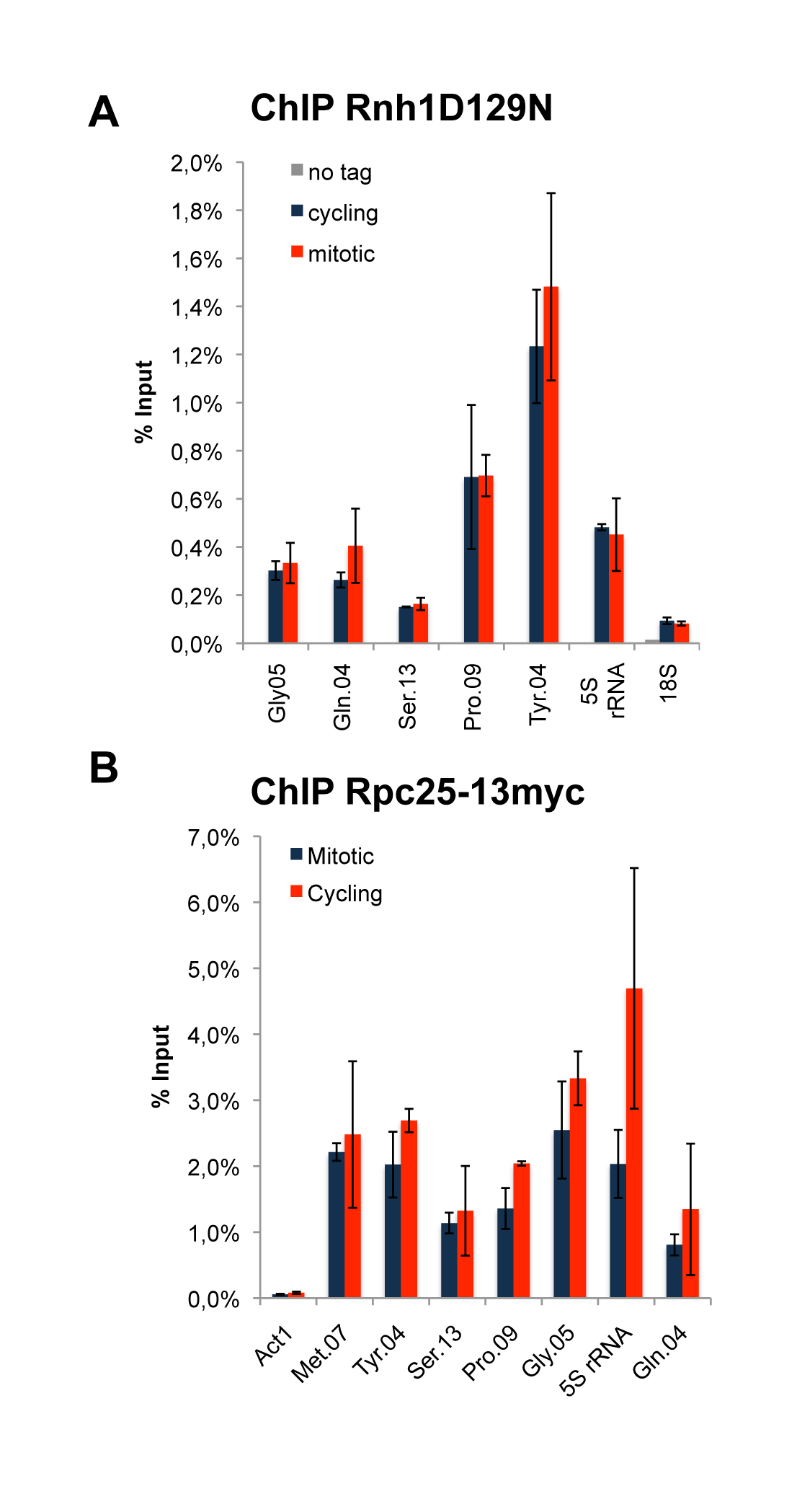

Supplement: Figure S7 — R-Loop form at Pol III-transcribed genes in early mitotic cells. nda3+ (cycling) and nda3KM311 (mitotic) cells were grown at the restrictive temperature of 17°C for 6 hours to synchronize cells prior to anaphase onset. ChIP-qPCR was performed to monitor the association of (A) Rnh1D129N-GFP or (B) Rpc25-13myc at the indicated loci (mean ± standard deviation from 3 biological replicates). (TIF) [file pgen.1004794.s007.tif]

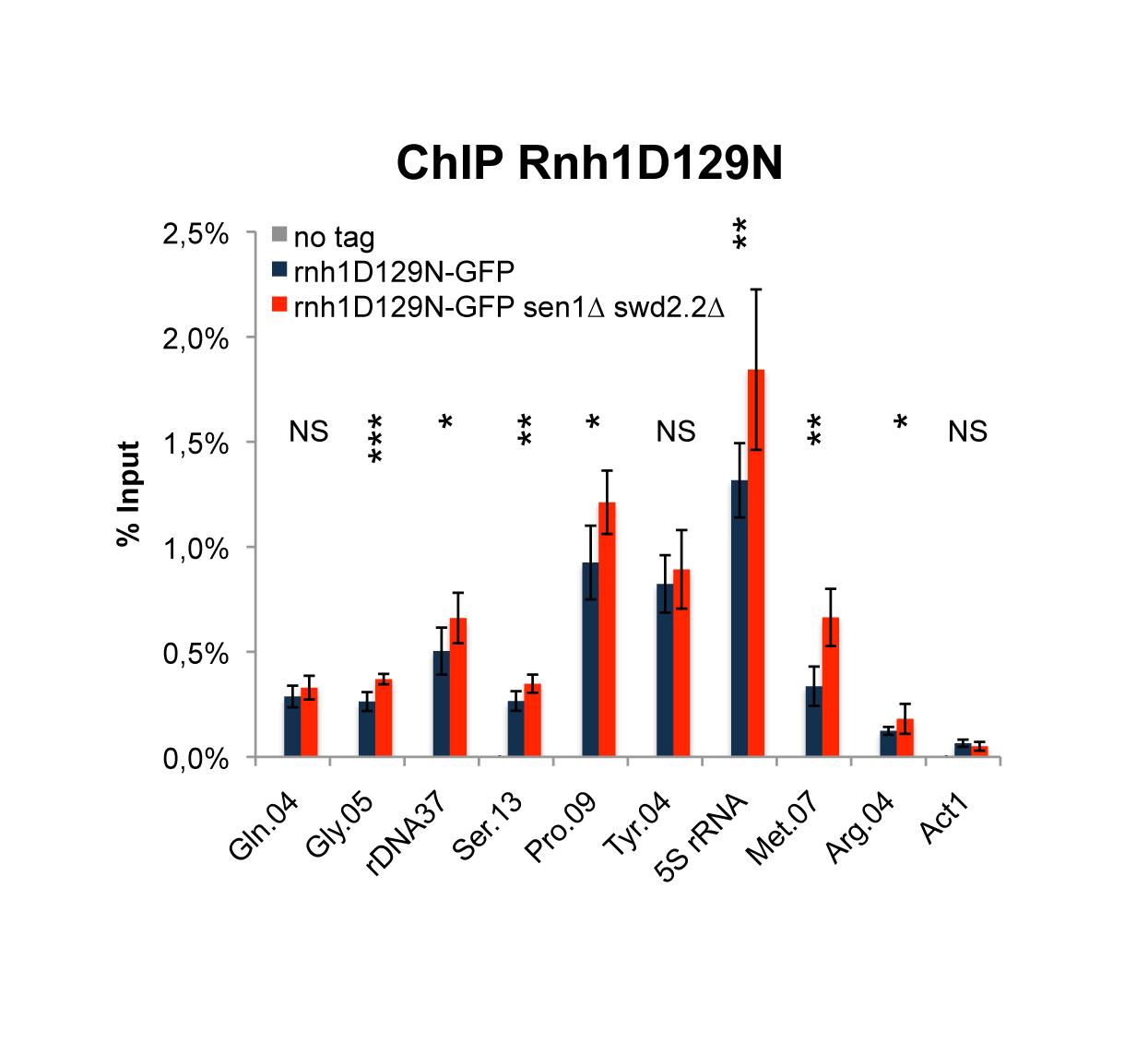

Supplement: Figure S8 — Lack of Swd2.2 and Sen1 results in a small but significant accumulation of R-Loops at Pol III-transcribed genes. ChIP qPCR of the indicated strains grown in cycling conditions at the indicated loci (mean ± standard deviation from 6 biological replicates. *P<0.05; **P<0.01; ***P<0.001 Wilcoxon - Mann Whitney). (TIF) [file pgen.1004794.s008.tif]

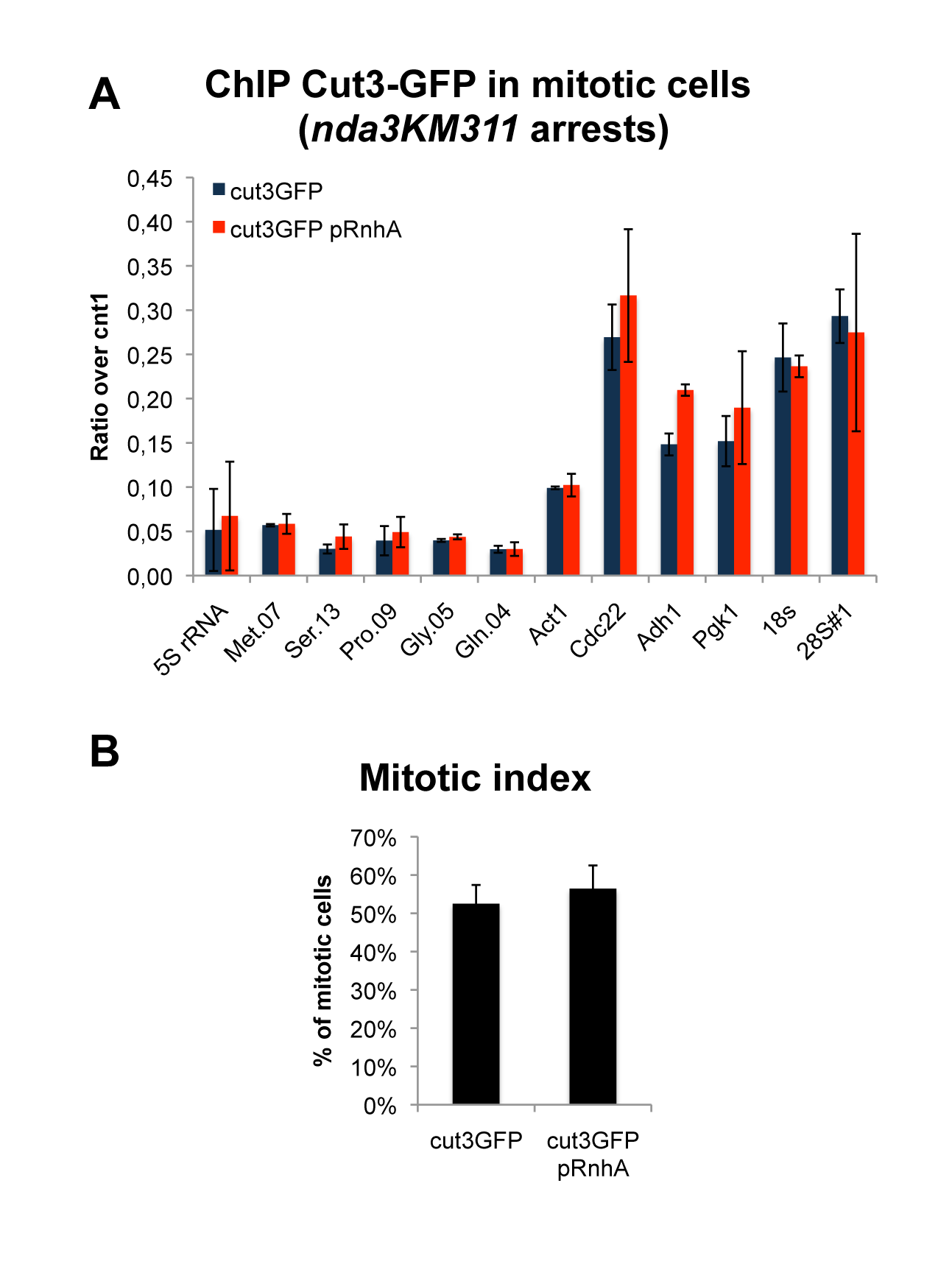

Supplement: Figure S9 — RnhA over-expression does not displace condensin from chromatin in mitotic cells. A. Cells carrying the cold-sensitive nda3-KM311 mutation were grown in minimum medium lacking thiamine for a minimum of 18 hours to drive the over-expression of RnhA by the nmt1 promoter and then shifted at 17°C for 6 hours to synchronize them prior to anaphase onset. ChIP-qPCR was then performed to analyze the association of GFP-tagged Cut3 with chromatin (mean ± standard deviation from 3 biological replicates). B. Mitotic indexes of the cell populations in A as established by scoring the number of Cut3-GFP-positive nuclei. (TIF) [file pgen.1004794.s009.tif]

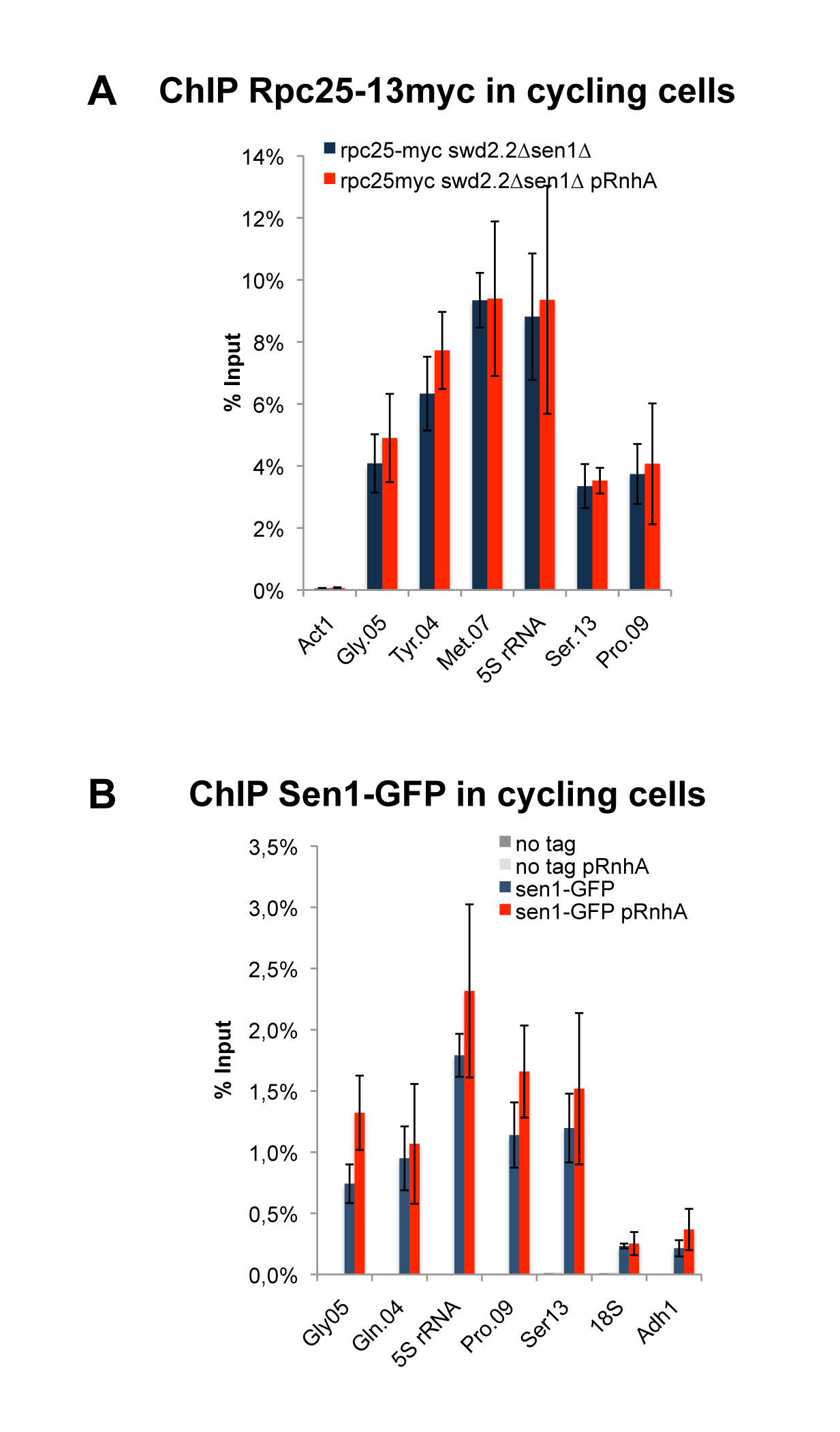

Supplement: Figure S10 — RnhA over-expression does not impact the association of RNA Pol III or Sen1 at Pol III-transcribed genes. A. ChIP qPCR of the indicated strains grown in cycling conditions in minimum medium at the indicated loci (mean ± standard deviation from 3 biological replicates). B. ChIP qPCR of the indicated strains grown in cycling conditions in minimum medium at the indicated loci (mean ± standard deviation from 3 biological replicates). (TIF) [file pgen.1004794.s010.tif]

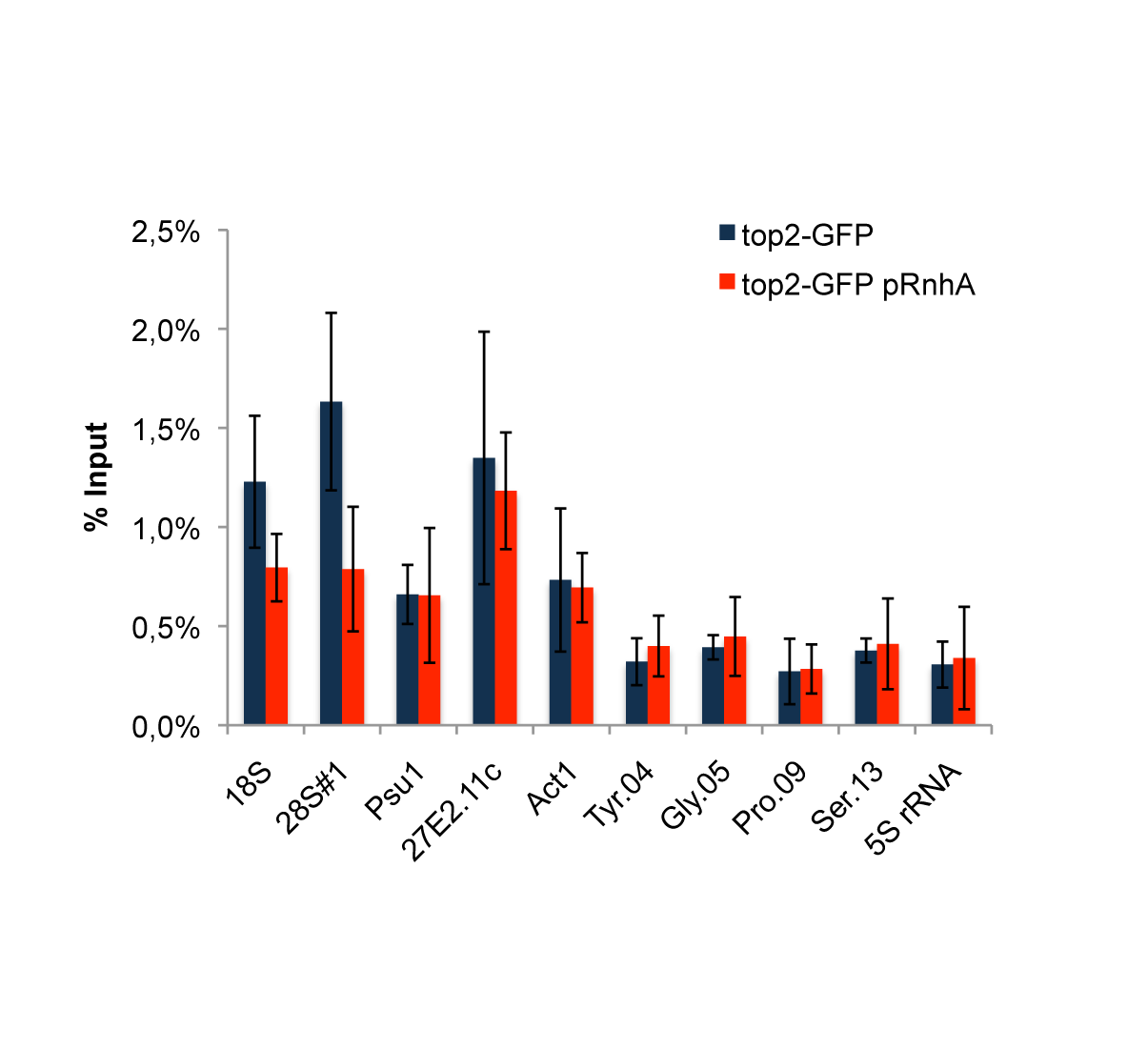

Supplement: Figure S11 — RnhA over-expression impacts the association of Top2 with chromatin differently at the rDNA and at Pol III-transcribed genes. ChIP qPCR of the indicated strains grown in cycling conditions in minimum medium at the indicated loci (mean ± standard deviation from 3 biological replicates). (TIF) [file pgen.1004794.s011.tif]

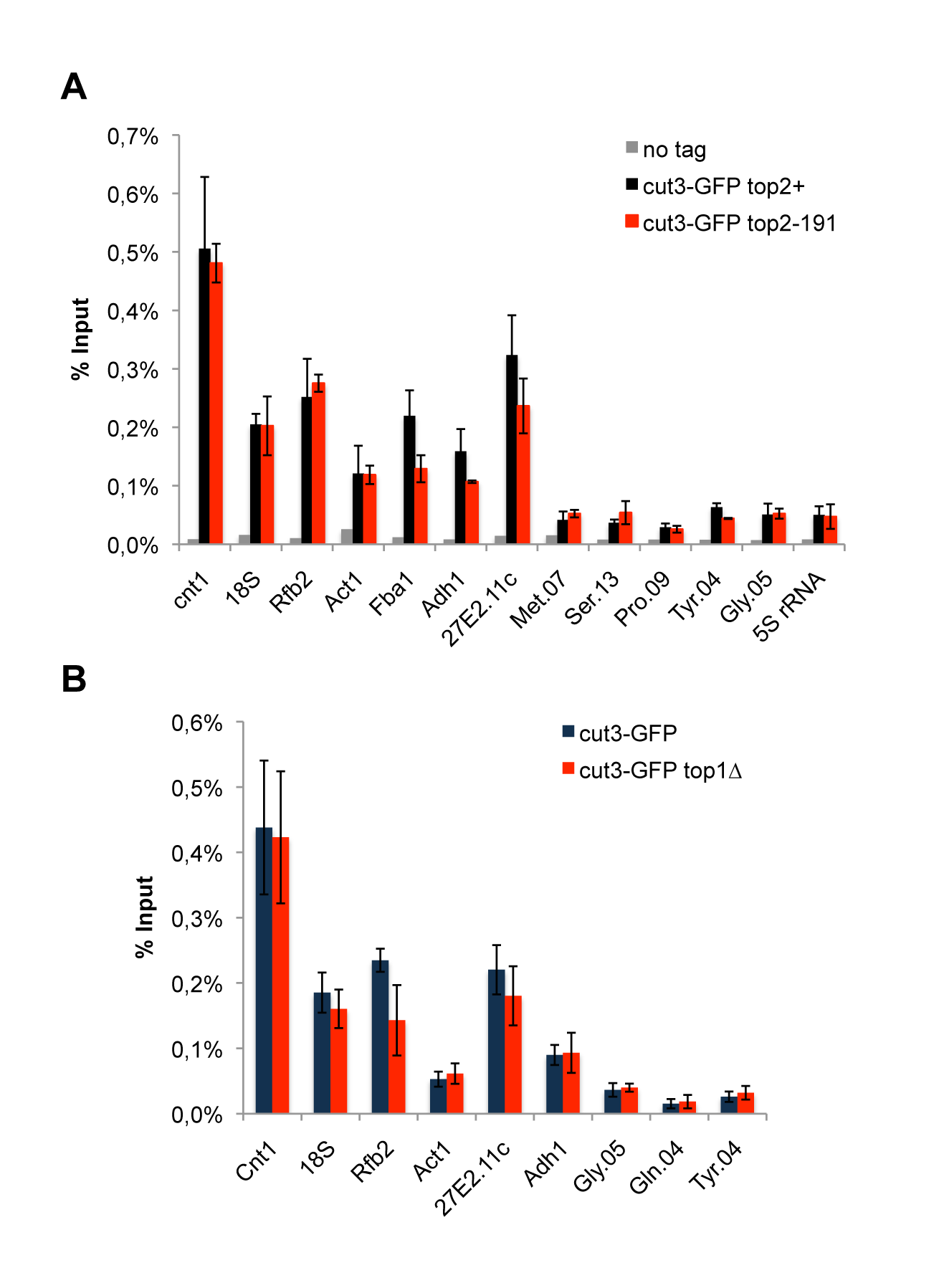

Supplement: Figure S12 — The association of condensin with chromatin is not significantly altered in the topoisomerase mutants top2-191 and top1Δ. A ChIP qPCR of the indicated strains grown in cycling conditions at the semi-restrictive temperature of 28°C at the indicated loci (mean ± standard deviation from 3 biological replicates). B. ChIP qPCR of the indicated strains grown in cycling conditions at the semi-restrictive temperature of 30°C at the indicated loci (mean ± standard deviation from 6 biological replicates). (TIF) [file pgen.1004794.s012.tif]
